# M2-polarized tumor-associated macrophages facilitated migration and epithelial-mesenchymal transition of HCC cells via the TLR4/STAT3 signaling pathway

**DOI:** 10.1186/s12957-018-1312-y

**Published:** 2018-01-16

**Authors:** Rong-Rong Yao, Jing-Huan Li, Rui Zhang, Rong-Xin Chen, Yan-Hong Wang

**Affiliations:** 1 Key Laboratory of Carcinogenesis and Cancer Invasion, Ministry of Education, Shanghai, 200032 China; 2 Liver Cancer Institute, Zhongshan Hospital, Fudan University, Shanghai, 200032 China

**Keywords:** Hepatocellular carcinoma, Epithelial-mesenchymal transition, Tumor-associated macrophages, Toll-like receptor 4

## Abstract

**Background:**

M2-polarized macrophages are tumor-associated-macrophages (TAMs), which are important contents of tumor-infiltrating immune cells. Toll-like receptor 4 (TLR4) is a molecular biomarker of tumor aggressiveness and poor prognosis. Toll-like receptors (TLRs) have important roles in the immune system and M2-polarized macrophages. However, the effects of TLR4 on M2-polarized macrophages in hepatocellular carcinoma (HCC) are unknown. Here, TLR4 expressed on HCC cells mediates the pro-tumor effects and mechanisms of M2-polarized macrophages.

**Methods:**

THP-1 cells were induced to differentiate into M2-like macrophages through treatments with IL-4, IL-13, and phorbol myristate acetate (PMA). We used the HCC cell lines SMMC-7721 and MHCC97-H cultured in conditioned medium from M2-like macrophages (M2-CM) to investigate the migration potential of HCC cells and epithelial-mesenchymal transition (EMT)-associated molecular genetics. Signaling pathways that mediated M2-CM-promoted HCC migration were detected using western blotting.

**Results:**

HCC cells cultured with M2-CM displayed a fibroblast-like morphology, an increased metastatic capability, and expression of EMT markers. TLR4 expression was markedly increased in M2-CM-treated HCC cells. TLR4 overexpression promoted HCC cell migration, and a TLR4-neutralizing antibody markedly inhibited HCC EMT in cells cultured with M2-CM. Furthermore, the TLR4/(signal transducer and activator of transcription 3 (STAT3) signaling pathway contributed to the effects of M2-CM on HCC cells.

**Conclusions:**

Taken together, M2-polarized macrophages facilitated the migration and EMT of HCC cells via the TLR4/STAT3 signaling pathway, suggesting that TLR4 may be a novel therapeutic target. These results improve our understanding of M2-polarized macrophages.

**Electronic supplementary material:**

The online version of this article (10.1186/s12957-018-1312-y) contains supplementary material, which is available to authorized users.

## Background

Hepatocellular carcinoma (HCC) is the primary liver cancer, which causing cancer-related death is the third of globally [1], in spite of recent advances in HCC diagnosis and treatment. The 5-year survival rate of HCC patients is still very low due to recurrence and metastasis, emphasizing the need to understand the molecular mechanisms of HCC recurrence and metastasis and to suggest novel therapeutic targets.

The important role of the immune microenvironment in HCC pathogenesis has been widely explored in recent years. M2-polarized macrophages are generally considered tumor-associated macrophages (TAMs) that sustain tumor progression and one of the primary tumor-infiltrating immune cells [2]. M2-polarized macrophages further tumor cell growth, invasion, and metastasis by secreting several cytokines [3–5] and stimulating specific signaling pathways, such as the transforming growth factor β1 (TGF-β1) [6] and the IL-10 signaling pathways [7]. Furthermore, according to several clinical studies, an increased number of TAMs in the tumor site frequently correlates with a poorer prognosis [8, 9]. However, the exact mechanisms by which M2-polarized macrophages modulate the migration potential of HCC are not completely defined.

Toll-like receptors (TLRs) are important receptors in the immune system and help to provide an advantageous microenvironment for cancer cells. But, the rigid roles of TLRs in cancer biology are still barely understood. TLR4 is overexpressed in macrophages and cancer cells, including colon cancer cells, lung cancer cells, and melanoma cells [10]. TLR4 is a cancer stem cell marker in HCC [11], and TLR4 expression increases tumor-initiating activity and chemoresistance during HCC development [12]. Recent research revealed an association between TLR4 expression and tumor aggressiveness and a poor prognosis for patients with HCC [13], although the mechanisms by which TLR4 promotes cancer progression are still unknown.

Therefore, we initially hypothesized that M2-polarized macrophages induce epithelial-mesenchymal transition (EMT) and accelerate the invasion and migration of HCC cells via a TLR4-dependent signaling pathway.

## Methods

### Cell lines and culture conditions

HCC cell lines (SMMC-7721 and MHCC97-H) were cultured in DMEM (GNM12800) medium, and THP-1 cells were maintained in RPMI 1640 (GNM31800) medium. Both of the mediums are supplemented with 10% fetal bovine serum (FBS, Gibco, USA), 50 U/ml streptomycin sulfate (Beyotime, China), and 50 U/ml penicillin (Beyotime, China). MHCC97-H cells were obtained from the Liver Cancer Institute, Zhongshan Hospital, Fudan University. The Shanghai Cell Bank of the Chinese Academy of Sciences provided THP-1 and SMMC-7721 cells. All cells were maintained at 37 °C in a humidified 5% CO_2_ atmosphere.

### PMA treatment for differentiating THP-1 cells into M2-polarized macrophages

THP-1 cell could be induced macrophage differentiation by phorbol myristate acetate (PMA, Sigma).THP-1 cells were cultured in RPMI 1640 complete medium with PMA treatment (320 nM/10^6^ cells) for 6 h, and then addition 20 ng/ml IL-13 and 20 ng/ml IL-4 incubated for 18 h to generate M2-polarized macrophages [14, 15]. CD68 expression in THP-1 cells and in induced M2-polarized macrophages was analyzed using a FACS Calibur flow cytometer. The differences in the relative expression levels of IL-10, TGF-β1, IL-12, and IL-23 between M2-polarized macrophages and macrophages were detected by quantitative real-time PCR (qRT-PCR).

### Collection of M2-CM and treatment of HCC cell lines

Macrophages and M2-polarized macrophages were washed three times with serum-free RPMI 1640 after treatment and cultured in RPMI 1640 supplemented with 10% FBS for 24 h. After culturing for 24 h, the fresh medium is replaced. The M2-like macrophage-conditioned medium (M2-CM) and control-conditioned medium (Control-CM) (RPMI 1640 with 10% FBS incubated at 37 °C in a humidified 5% CO_2_ atmosphere for 24 h) were collected by centrifugation at 3000 rpm for 15 min for further use.

HCC cell lines (SMMC-7721 and MHCC97-H) were divided into the experimental group and the control group. Cells in the experimental group were cultured in DMEM containing 10% FBS and M2-CM for 48 h, and cells in the control group were cultured in DMEM with 10% FBS and Control-CM. The volumetric ratio of DMEM and M2-CM/Control-CM was 1:1.

### Wound-healing assay and transwell migration assay

The detailed methods for the wound-healing and the transwell assays are described in a previous study [3]. Photographs of the wound-healing assay were captured at 0 and 48 h using a phase-contrast microscope, and the distance traveled by migrating cells at the wound front was measured. We defined L_0 h_–L_48 h_ as the length of wound healing. Experiments were performed in triplicate.

Approximately 5 × 10^4^ HCC cells were seeded in the upper chamber (8.0-μm pore size, Corning) for the transwell migration assay. 600 μl of culture medium were added to the lower compartment. Cells were cultured for 24 h then were fixed in 4% paraformaldehyde and stained with 0.1% crystal violet. The amount of cells was counted under a microscope (× 100). Experiments were performed in triplicate.

### Western blot

Detailed protein extraction and western blot methods are described in a previous study [3]. Non-specific binding was blocked by incubating the membranes with 5% bovine serum albumin (BSA, Sigma) for 2 h before an overnight incubation with primary antibodies against E-cadherin (ab40772, diluted 1:5000), N-cadherin (ab76011, diluted 1:5000), vimentin (ab92547, diluted 1:1000), TLR4 (ab22048, diluted 1:400), STAT3 (Cell Signaling Technology, #4904, diluted 1:500), p-STAT3 (Cell Signaling Technology, #9415, diluted 1:500), ERK (Cell Signaling Technology, #9102, diluted 1:500), p-ERK (Cell Signaling Technology, #4370, diluted 1:500), Ikkα, Ikkβ, p-Ikkα/β (Cell Signaling Technology, #9936, diluted 1:1000), and beta-actin (AA128, diluted 1:5000). Samples were incubated with a secondary antibody (Jackson ImmunoResearch, diluted 1:5000), and the blots of target proteins were detected using an enhanced chemoluminescence substrate (Beyotime, P0018).A densitometry of scanned blots were analysis using Image Lab software. Experiments were performed in triplicate.

### Real-time PCR

Total RNA was extracted using Trizol (Invitrogen, Carlsbad, CA, USA). Using qRT-PCR with the SYBR Green Master Mix (Themo, USA), we analyzed the expression of mRNAGlyceraldehyde-3-phosphatedehydrogenase (GAPDH) using as endogenous references. Table 1 lists the primers used in the present study. Experiments were performed in triplicate.

**Table 1:** The primers were used in the experiments

IL-10 Forword 5′-TCTCCGAGATGCCTTCAGCAGA-3′
Reverse 5′-TCAGACAAGGCTTGGCAACCCA-3′
TGF-β1 Forword 5′-TACCTGAACCCGTGTTGCTCTC-3′
Reverse 5′-GTTGCTGAGGTATCGCCAGGAA-3′
IL-23 Forword 5′-GAGCCTTCTCTGCTCCCTGATA-3′
Reverse 5′-GACTGAGGCTTGGAATCTGCTG-3′
IL-12A Forword 5′-TGCCTTCACCACTCCCAAAACC-3′
Reverse 5′-CAATCTCTTCAGAAGTGCAAGGG-3′
TLR4 Forword 5′-CCCTGAGGCATTTAGGCAGCTA-3′
Reverse 5′-AGGTAGAGAGGTGGCTTAGGCT-3′
E-cadherin Forword 5′-TGATTCTGCTGCTCTTGCTG-3′
Reverse 5′-CTCTTCTCCGCCTCCTTCTT-3′
N-cadherin Forword 5′-CGTGAAGGTTTGCCAGTGT-3′
Reverse 5′-CAGCACAAGGATAAGCAGGA-3′
Vimentin Forword 5′-AGAGAACTTTGCCGTTGAAGC-3′
Reverse 5′-ACGAAGGTGACGAGCCATT-3′
GAPDH Forword 5′-GTCTCCTCTGACTTCAACAGCG-3′
Reverse 5′-ACCACCCTGTTGCTGTAGCCAA-3′

### Statistical analysis

The analysis was conducted with SPSS19.0 software. Student’s *t* test was used for comparison between two groups, and variance (ANOVA) was used for comparisons among multiple groups. All data are expressed as the means ± standard errors of the means (SEM) from at least three separate experiments. *P < 0.05* was considered statistically significant.

## Results

### HCC cells exhibit a fibroblast-like morphology after treatment with M2-CM

**Fig. 1:**
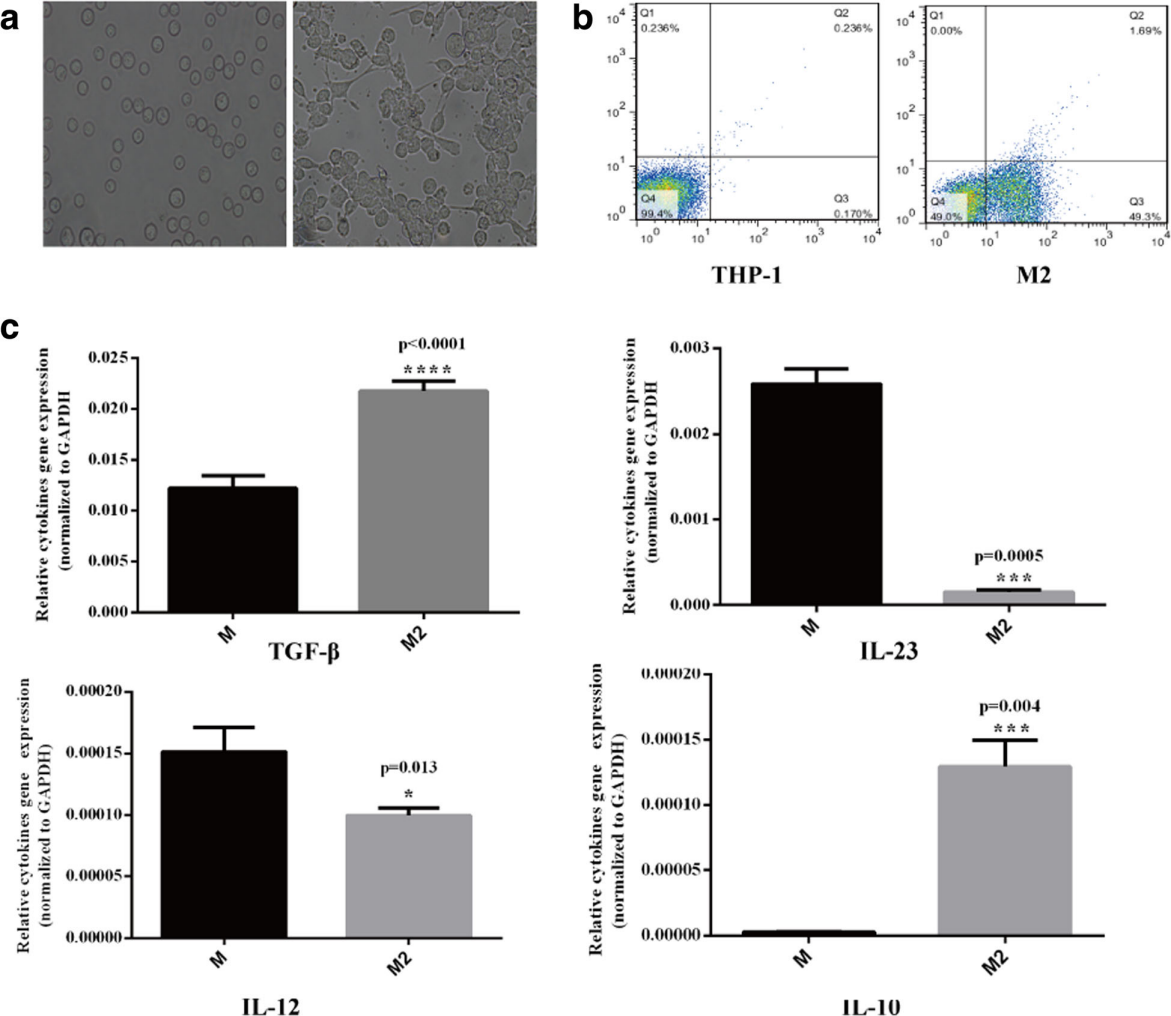
THP-1 cells were successfully differentiated into M2-polarized macrophages. **a** Images of THP-1 cultured under normal conditions (left) or with PMA (320 nM) for 6 h and subsequently cultured with IL-4 (20 ng/ml) and IL-13 (20 ng/ml) for 18 h (right) (× 200). **b** Flow cytometry analysis: normal THP-1 cells (left) and PMA + IL-4 + IL-13-treated THP-1 cells (right) exhibit significant differences in CD68 expression (a marker of macrophage differentiation). **c** M2 markers were detected in native and M2 macrophages using RT-PCR. Compared with native macrophages, M2-polarized macrophages exhibit the IL-12^low^, IL-23^low^, IL-10^high^, and TGF-β^high^ phenotype

**Fig. 2:**
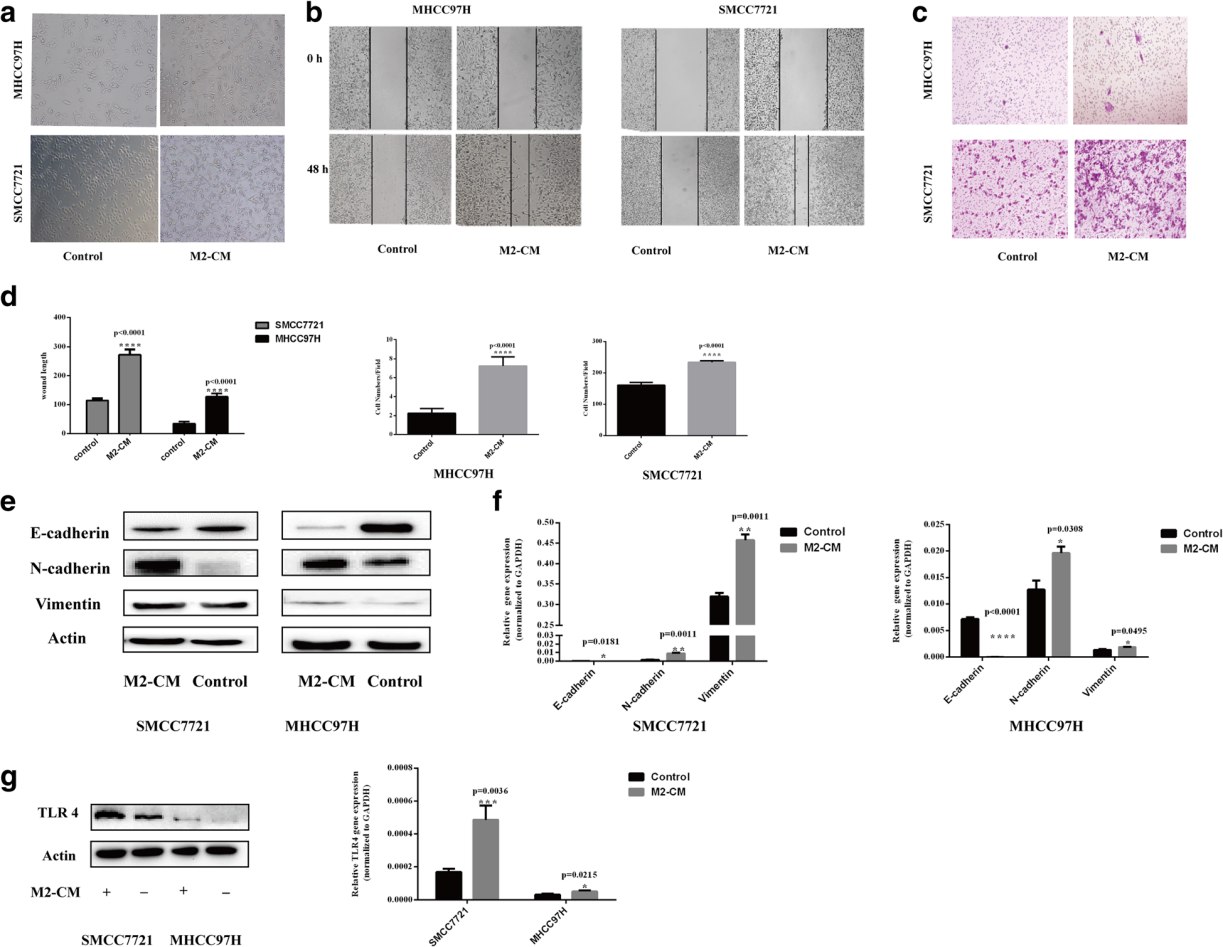
M2-CM increased the malignant properties of HCC cells and induced TLR4 activation. **a** M2-CM increased the number of HCC cells with the fibroblast-like morphology (magnification, × 100). **b** Wound-healing assay. Wound closure was delayed in M2-CM-treated MHCC97H and SMMC7721 cells compared with in the control group at 48 h (magnification, × 50). **c** Transwell migration assays. The number of cells passing through the upper chamber was counted in four fields (magnification, × 100). **d** Analysis of the results of the wound-healing assay and transwell migration assay. **e**–**f** M2-CM promoted EMT in HCC cells. The expression of EMT markers E-cadherin, N-cadherin, and vimentin in M2-CM-stimulated HCC cells, and the control group was analyzed using western blots and RT-PCR. **g** M2-CM induced TLR4 activation in HCC cells. The expression of TLR4 on HCC cells in M2-CM and control cells was detected using western blots and RT-PCR. Date are shown as the means ± SD (^*^P < 0.05, ^**^P < 0.01, ^***^P < 0.001, ^****^P < 0.0001). The data represent at least three independent experiments

We induced THP-1 cells to differentiate into M2-polarized macrophages as described above and verified the M2-polarized macrophage phenotype by examining the cell morphology and cytokine and surface marker expression (Fig. 1a–c). After culturing with M2-CM, MHCC97H, and SMCC7721, two HCC cell lines with different metastatic potentials exhibited morphologically distinct features from the typical epithelial appearance of control cells. Cells were spindle-shaped with less cell-cell adhesion and increased pseudopodia formation (Fig. 2a).

### M2-CM promotes the migration and EMT of HCC cells

We investigated the migration potential of HCC cells in vitro following culture with M2-CM. M2-CM-treated HCC cells migrated a much longer distance than control cells in wound-healing assays (Fig. 2b and d, *P* < 0.001). In the transwell migration assays, a greater number of HCC cells treated with M2-CM had migrated compared with the number of migrating control cells (Fig. 2c, d, *P* < 0.001). Western blotting and qRT-PCR were performed to further investigate the expression of EMT-associated molecular markers because of the altered morphology of HCCs after M2-CM treatment. Western blots revealed higher levels of expression of mesenchymal marker proteins, including vimentin and N-cadherin, in HCC cells cultured with M2-CM. Levels of E-cadherin, a marker of epithelial cells, were decreased in the M2-CM-treated groups compared with the control (Fig. 2e). These results were supported by RT-PCR results showing higher levels of vimentin and N-cadherin mRNAs in the experimental group and lower levels of E-cadherin mRNA (Fig. 2f). Therefore, culture with M2-CM increased the expression of several mesenchymal markers and the migration of HCC cells in vitro.

### M2-CM increases TLR4 expression in HCC cells

RT-PCR and western blots were used to compare TLR4 expression levels in the M2-CM-treated and control groups to reveal the correlation of TLR4 expression with M2-CM-triggered HCC malignancy. Based on the RT-PCR results, M2-CM significantly increased TLR4 expression in HCC cells (Fig. 2g). Western blot data also confirmed that M2-CM increased the expression of TLR4 in HCC cells (Fig. 2g).

### TLR4 overexpression further promotes the malignant properties of HCC cells cultured with M2-CM

**Fig. 3:**
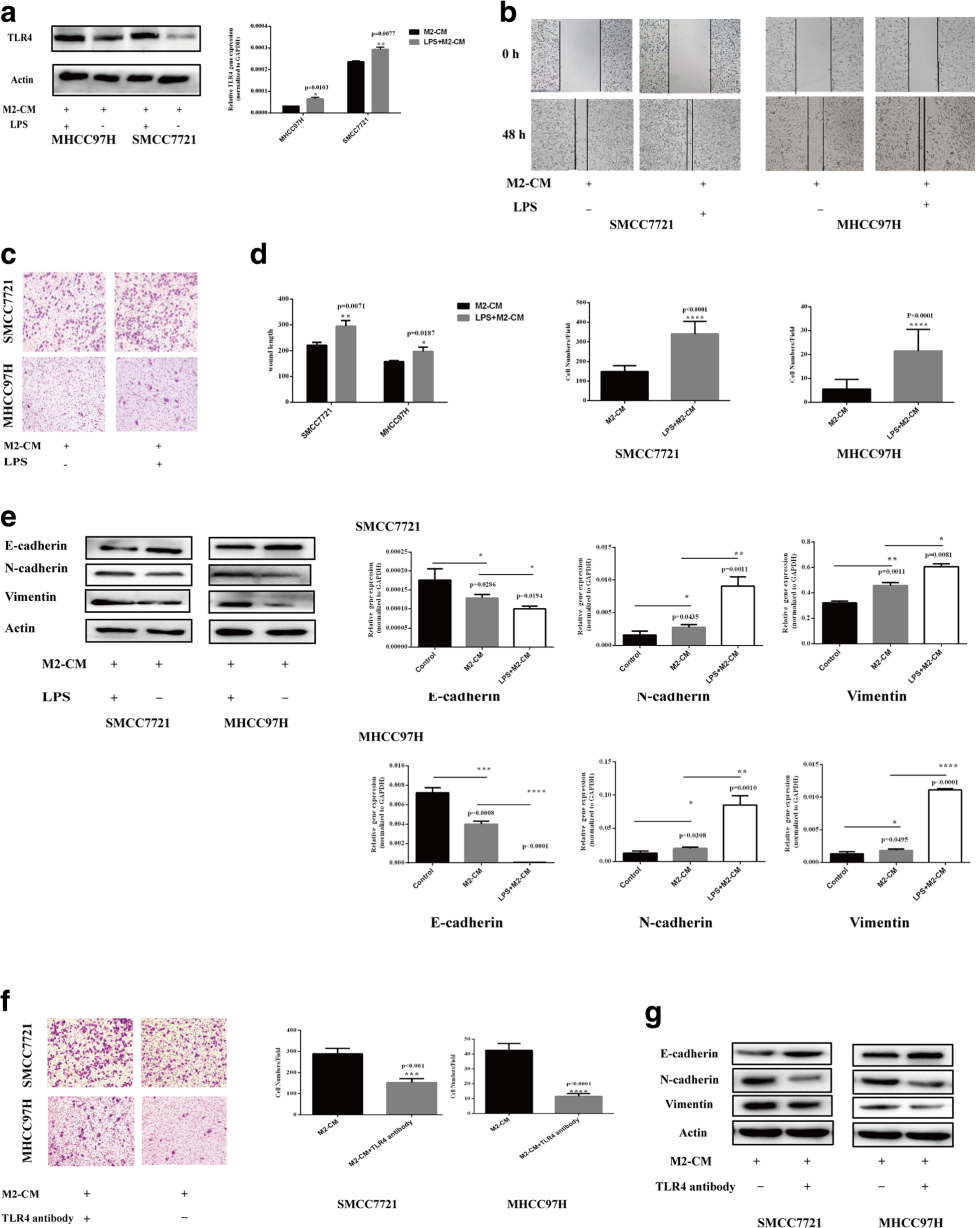
TLR4 played a crucial role in the M2-CM-induced increase in the malignant properties of HCC cells. **a** LPS successfully upregulated TLR4 expression in HCC cells. TLR4 expression in HCC cells was detected using western blots and RT-PCR in the M2-CM plus LPS and M2-CM groups. TLR4 upregulation promoted the M2-CM-induced migration of HCC cells. **b** The distance traveled by migrating HCC cells in the M2-CM + LPS and M2-CM alone groups was measured using a wound-healing assay (× 50). **c** Cell migration was determined using the transwell assay (× 100). **d** Analyses of the results of the transwell migration and wound-healing assays. **e** TLR4 upregulation promoted EMT in cells cultured with M2-CM. The expression of E-cadherin, N-cadherin, and vimentin in the control, M2-CM + LPS, and M2-CM groups was analyzed using western blotting and RT-PCR. **f** A TLR4 neutralizing antibody inhibited M2-CM-induced HCC cell migration. Cell migration was determined in the M2-CM + TLR4 antibody and M2-CM groups using the transwell assay (× 100). **g** The TLR4 neutralizing antibody inhibited M2-CM-induced EMT. The expression of E-cadherin, N-cadherin, and vimentin in the M2-CM + TLR4 antibody and M2-CM groups was analyzed using western blots. Data are shown as the means ± SD (*P < 0.05, **P < 0.01, ***P < 0.001, ****P < 0.0001). The data represent at least three independent experiments

We investigated the effects of treatment with the TLR4 agonist LPS, which upregulates TLR4 expression, and the M2-CM treatment to elucidate the role of TLR4 in HCC cell malignancy [11, 16, 17]. As shown in Fig. 3a, treatment with both M2-CM and LPS increased the levels of the TLR4 protein and mRNA in MHCC97H and SMCC7721 cells. In the migration assays, greater numbers of cells treated with M2-CM plus LPS migrated through the membrane compared with cells treated with M2-CM alone (Fig. 3c, d, *P* < 0.001). Wound-healing assays revealed significantly enhanced motility of HCC cells following treatment with both LPS and M2-CM (Fig. 3b, d, *P* < 0.05). The RT-PCR and western blot results for vimentin, N-cadherin, and E-cadherin expression further supported the finding that upregulated TLR4 expression increased the malignant properties of HCC cells cultured with M2-CM (Fig. 3e). Moreover, the LPS treatment (without M2-CM) did not obviously increase the malignant properties of control HCC cells (Additional file 1: Figure S1).

### A TLR4-neutralizing antibody markedly inhibits M2-CM-induced EMT and migration

A TLR4 neutralizing antibody (10 μg/ml) was added to further investigate the effect of TLR4 on the crosstalk between M2-CM and HCC cells. Compared with control cells, fewer HCC cells treated with a TLR4-neutralizing antibody migrated toward M2-CM in the transwell migration assays (Fig. 3f). According to the western blot results, in cells cultured with M2-CM, the TLR4 neutralizing antibody reversed the reduction in E-cadherin levels and the increase in N-cadherin and vimentin levels compared to the control group (Fig. 3g).

### TLR4/STAT3 pathways are involved in M2-CM-mediated enhancement of the malignancy of HCC cell lines

**Fig. 4:**
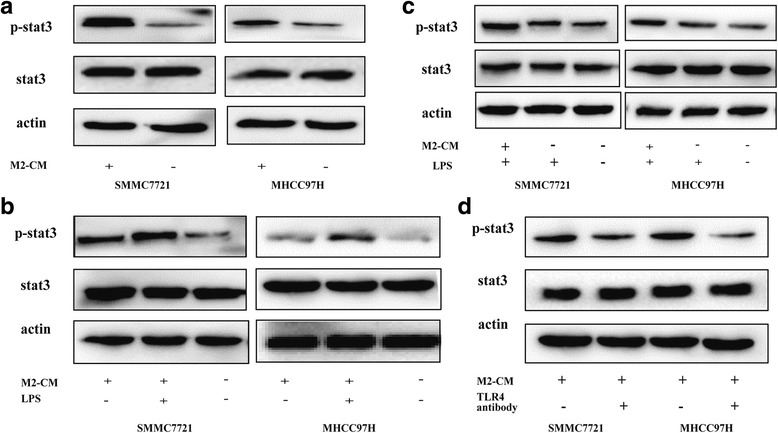
M2-CM activated the TLR4/STAT3 pathway in HCC cells. **a** Levels of p-STAT3 and total STAT3 in the M2-CM and control groups were analyzed using western blotting. **b** TLR4 overexpression increased the p-STAT3/STAT3 ratio in M2-CM-treated HCC cells. Level of p-STAT3 and total STAT3 in the control, M2-CM + LPS, and M2-CM groups. **c** LPS alone did not significantly increase p-STAT3 levels in HCC cells. Levels of the p-STAT3 and STAT3 proteins in the control, LPS, and M2-CM + LPS groups. **d** The TLR4 neutralizing antibody inhibited the M2-CM-induced increase in the p-STAT3/STAT3 ratio in HCC cells. Levels of p-STAT3 and total STAT3 were analyzed using western blotting

We elucidated the signaling pathways involved in the effect of M2-polarized macrophages on promoting malignancy in HCC cells. Levels of the Ikkα, Ikkβ, p-Ikkα/β, STAT3, p-STAT3, ERK, and p-ERK proteins were detected using western blot analyses. Western blotting revealed elevated levels of STAT3 phosphorylation in HCC cells after treatment with M2-CM (Fig. 4a). Higher levels of p-STAT3 were also observed in the M2-CM + LPS group than in the M2-CM only group following the upregulation of TLR4 (Fig. 4b), and treatment with LPS alone did not obviously increase p-STAT3 levels in HCC cells (Fig. 4c). However, significant increases in Ikk and ERK phosphorylation were observed (Additional file 2: Figure S2). The application of TLR4 neutralizing antibodies markedly inhibited STAT3 phosphorylation in HCC cells (Fig. 4d). Based on these data, the TLR4/STAT3 pathway was involved in the M2-CM-mediated enhancement of the malignancy of HCC cell lines.

## Discussion

Recent literature highlights the possibility of crosstalk between tumor cells and non-tumor cells in the tumor microenvironment. It is well known that the critical roles of stromal cells are regulating tumorigenesis, EMT, tumor metastasis, and invasion [18]. In the present study, M2-CM promoted HCC migration by upregulating TLR4 expression and stimulating the TLR4/STAT3 signaling pathway in HCC cells. Our study provides the first evidence that activation of the TLR4/STAT3 pathways take part in M2-polarized macrophage-induced HCC invasion and metastasis.

M2-polarized macrophages are main components of tumor-infiltrating stromal cells and are key factors contributing to tumor progression. M2-polarized macrophages exhibited increased migration, which is related to EMT in HCC cells. Consistent with these data, M2-polarized macrophages were reported to further tumor cell growth, invasion, and metastasis by secreting certain chemokines, growth factors, cytokines, and matrix metalloproteases, such as TGF-β1, IL-10, IL-6, and IL-8. Based on our data and previous findings, these factors are primarily derived from M2-polarized macrophages rather than M1-polarized macrophages. IL-6 promotes the expansion of HCC stem cells [19], and IL-10 promotes EMT in pancreatic cancer cells [7]. TGF-β1 induces cancer stem cell-like properties in HCC and glioma [3–6].IL-8 and IL-6 enhance the invasion of LoVo cells [15]. The crosstalk between M2-polarized macrophages and cancer is complex, and it is involved in each step of HCC development. According to Kurahara H et al., M2-polarized macrophages facilitate angiogenesis, tissue remodeling and repair [20]. TAMs were recently shown to induce cancer stem cell-like properties and chemoresistance in HCC cells [19, 21]. In addition, tumor-infiltrating macrophages located in the invasive margins of cancer are a beneficial prognostic marker for various cancers [9, 22].

Although many studies have reported a role for M2-polarized macrophages in tumor metastasis, the underlying mechanism has not been clearly delineated. In the present study, M2-polarized macrophages upregulated TLR4 expression in HCC cells and activated the STAT3 signaling pathway downstream of TLR4, which may be one of the primary mechanisms for promoting metastasis. Previous research also suggested the importance of TLR4 in HCC. Recently, Liu WT et al. were the first to show that TLR4 facilitates the migration and invasion of HCC cells and functions as a cancer stem cell marker, further emphasizing the importance of TLR4 in HCC metastasis and recurrence [11]. Meanwhile, some researchers have revealed roles for TLR4-activated signaling pathways in cancer. Chih-Cheng Hsiao and colleagues reported the involvement of the Akt and mitogen-activated protein kinase (MAPK) pathways in LPS-enhanced TLR4 activation, along with increased cell proliferation, NOS expression, and chemoresistance in HepG2 cells [16]. Activation of the NF-κB signaling pathway downstream of TLR4 signaling increased the proliferation and invasion of HCC [23]. In addition to these signaling pathways, the classical TLR4/MyD88 signaling pathway is also responsible for cancer progression [24] (Additional file 3).

TLR4 promotes cancer progression by activating several signaling pathways, but M2-CM specifically activated the STAT3 signaling pathway in the present study. STAT3, a pivotal member of the signal transducer and activator of transcription family, has been detected in lots of HCC samples. STAT3 is correlated with invasiveness and a poor prognosis. We further examined the role of STAT3-mediated TLR4 signaling and observed lower p-STAT3/STAT3 ratios when HCC cells were treated with M2-CM plus a TLR4 neutralizing antibody. We upregulated TLR4 expression levels in HCC cells using M2-CM plus LPS, a TLR4 agonist, as described previously [17, 25]. Upon stimulation with M2-CM, higher p-STAT3/STAT3 ratios were observed in LPS-treated HCC cells than in control cells. Furthermore, LPS alone did not significantly increase p-STAT3 levels in HCC cells, suggesting that LPS did not interfere with STAT3 phosphorylation, in contrast to some previous reports. The main reason may be related to the timing of LPS stimulation, which is consistent with recent studies showing that LPS increases the levels of some proteins in a time-dependent manner [23, 25]. Therefore, we postulate that STAT3 functions downstream of TLR4 signaling. TLR4/STAT3 formed a critical axis that was successively activated by M2-polarized macrophages in HCC cells.

In the present study, we did not determine which cytokines secreted by M2-polarized macrophages activated TLR4/STAT3 signaling in HCC cells. However, cytokines secreted by M2-polarized macrophages modulate the function of the TLR4/STAT3 signaling pathway and act as a bridge between macrophages and HCC cells. The present study had several limitations. Native and M1-polarized macrophages were not used as negative control groups in any of the experiments performed in this study, as our study focused on the crosstalk between M2-polarized macrophages and HCC. Moreover, in the tumor microenvironment, TAMs polarization was M2 phenotype in several studies.

## Conclusions

Based on our results, M2-polarized macrophages facilitated the migration and EMT of human HCC cells by activating the TLR4/STAT3 signaling pathway. Our study highlights the importance of targeting the immune microenvironment as a mechanism to inhibit HCC recurrence and metastasis. TLR4 and its cognate signaling pathways might represent potential targets of therapeutics against HCC.

## Additional files

Additional file 1: Figure S1. Cells treated with LPS in the absence of M2-CM did not exhibit increased migration. (A) Cell migration in the control and LPS only groups was determined using the transwell assay (× 100). (B) The distance traveled by migrating HCC cells in the control and LPS only groups was measured using a wound-healing assay (× 50). (C) Analysis of the data from the transwell migration assay. (TIFF 16142 kb)

Additional file 2: Figure S2. Levels of the p-ERK, p-Ikkα/β, total STAT3, ERK, Ikkα, and Ikkβ proteins were detected using western blotting. (A) Levels of the p-STAT3 and STAT3 proteins in the control, LPS, and M2-CM + LPS groups. (B) Levels of the p-ERK and ERK proteins in the control, M2-CM + LPS, and M2-CM groups. (C) Levels of the p-Ikkα/β, Ikkα, and Ikkβ proteins in the control, M2-CM + LPS, and M2-CM groups. (TIFF 601 kb)

Additional file 3: Figure S3. Language edit certification. (JPEG 147 kb)
