# Supplementary figures and images for: M2-polarized tumor-associated macrophages facilitated migration and epithelial-mesenchymal transition of HCC cells via the TLR4/STAT3 signaling pathway
Source: World J Surg Oncol. 2018 Jan 16;16:9. doi: 10.1186/s12957-018-1312-y (PMC5771014; doi:10.1186/s12957-018-1312-y)

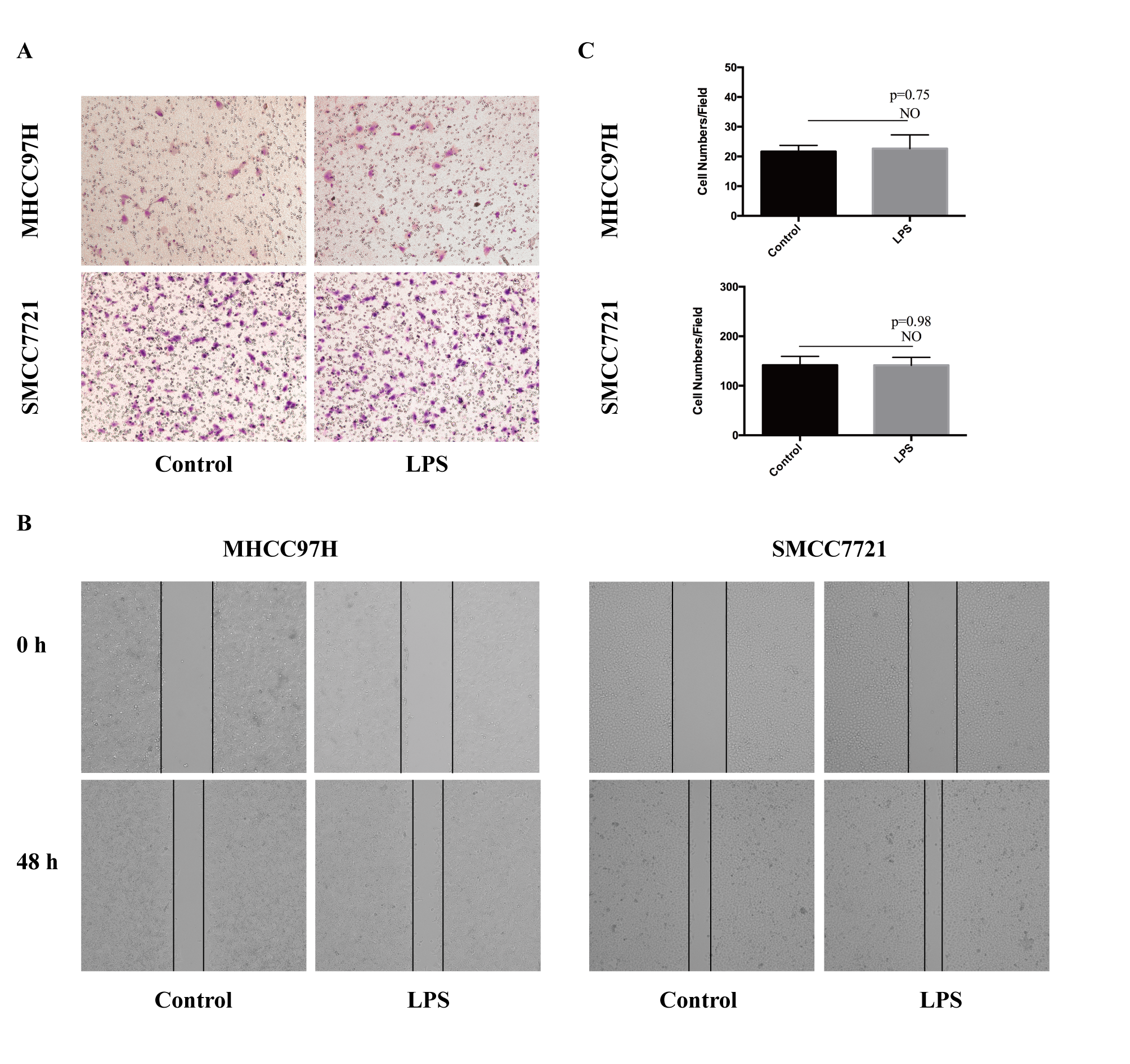

Supplement: Supplementary file 1 — Cells treated with LPS in the absence of M2-CM did not exhibit increased migration. (A) Cell migration in the control and LPS only groups was determined using the transwell assay (× 100). (B) The distance traveled by migrating HCC cells in the control and LPS only groups was measured using a wound-healing assay (× 50). (C) Analysis of the data from the transwell migration assay. (TIFF 16142 kb) [file 12957_2018_1312_MOESM1_ESM.tif]

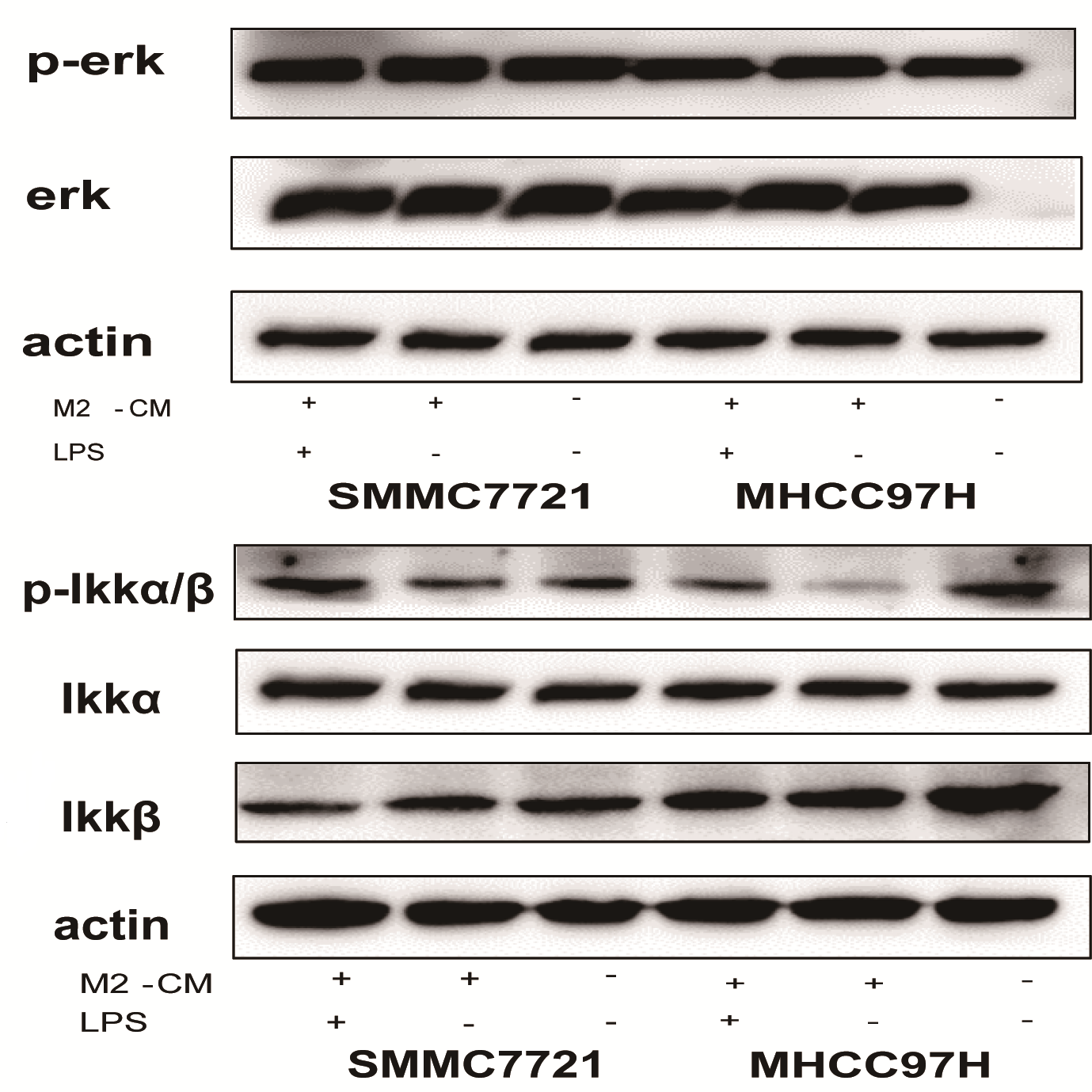

Supplement: Supplementary file 2 — Levels of the p-ERK, p-Ikkα/β, total STAT3, ERK, Ikkα, and Ikkβ proteins were detected using western blotting. (A) Levels of the p-STAT3 and STAT3 proteins in the control, LPS, and M2-CM + LPS groups. (B) Levels of the p-ERK and ERK proteins in the control, M2-CM + LPS, and M2-CM groups. (C) Levels of the p-Ikkα/β, Ikkα, and Ikkβ proteins in the control, M2-CM + LPS, and M2-CM groups. (TIFF 601 kb) [file 12957_2018_1312_MOESM2_ESM.tif]

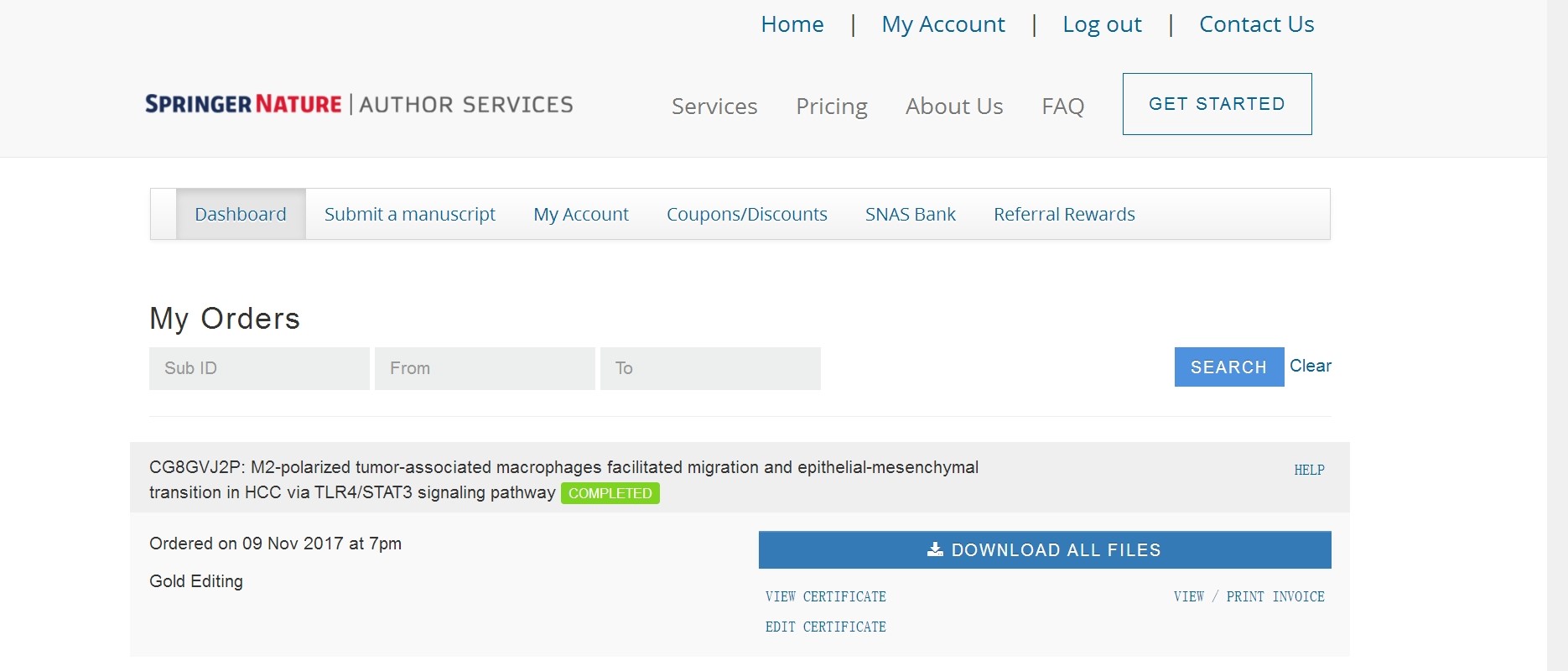

Supplement: Supplementary file 3 — Language edit certification. (JPEG 147 kb) [file 12957_2018_1312_MOESM3_ESM.jpg]
